# Validation of the English Version of the 14-Item Mediterranean Diet Adherence Screener of the PREDIMED Study, in People at High Cardiovascular Risk in the UK

**DOI:** 10.3390/nu10020138

**Published:** 2018-01-28

**Authors:** Angeliki Papadaki, Laura Johnson, Zoi Toumpakari, Clare England, Manmita Rai, Stu Toms, Chris Penfold, Itziar Zazpe, Miguel A. Martínez-González, Gene Feder

**Affiliations:** 1 Centre for Exercise, Nutrition and Health Sciences, School for Policy Studies, University of Bristol, 8 Priory Road, Bristol BS8 1TZ, UK; laura.johnson@bristol.ac.uk (L.J.); z.toumpakari@bristol.ac.uk (Z.T.); clare.england@bristol.ac.uk (C.E.); 2 NIHR Bristol Biomedical Research Centre (Nutrition Theme), University Hospitals Bristol NHS Foundation Trust and University of Bristol, Lower Maudlin Street, BristolBS1 2LY, UK; manmita.rai@bristol.ac.uk (M.R.); stu.toms@bristol.ac.uk (S.T.); chris.penfold@bristol.ac.uk (C.P.); 3 Department of Nutrition, Food Sciences and Physiology, University of Navarra, C/Irunlarrea 1, 31008 Pamplona, Spain; izazpe@unav.es; 4 Department of Preventive Medicine and Public Health, School of Medicine, University of Navarra, C/Irunlarrea 1, 31008 Pamplona, Spain; mamartinez@unav.es; 5 Department of Nutrition, Harvard TH Chan School of Public Health, Boston, MA 02115, USA; 6 Centre for Academic Primary Care, Bristol Medical School, Population Health Sciences, University of Bristol, Canynge Hall, 39 Whatley Road, BristolBS8 2PS, UK; gene.feder@bristol.ac.uk

**Keywords:** Mediterranean diet, Mediterranean diet score, cardiovascular risk, validity, accuracy, reliability

## Abstract

The aim of this study was to examine the validity of the English version of the *PREvencion con DIetaMEDiterranea* (PREDIMED) 14-item Mediterranean Diet Adherence Screener (MEDAS), a brief questionnaire assessing adherence to the Mediterranean diet (MedDiet), which was used in the PREDIMED trial for assessment and immediate feedback. This instrument (MEDAS) was administered to 96 adults with a high cardiovascular risk (66% women, mean age 68.3 ± 6.0 years), recruited from general practices in Bristol, UK. Participants then completed a 3-day estimated food record, and the MEDAS was administered again one month later. A MedDiet score (range = 0–14) was calculated from the MEDAS’ administrations and food record to assess concurrent validity and test-retest reliability. Predictive validity was assessed by examining the association of the MEDAS-derived score with cardiometabolic risk factors and dietary intakes derived from the food records. The MEDAS-derived MedDiet score was higher by 1.47 points compared to food records (5.47 vs.4.00, *p* < 0.001), correlated moderately with the record-derived score (*r* = 0.50, *p* < 0.001; ICC = 0.53, *p* < 0.001) and there was borderline fair agreement between the two methods (*κ* = 0.19, 95% CI 0.07–0.31, *p* = 0.002; 95% limits of agreement −2.2, 5.1). Exact agreement within score categories and gross misclassificationwere 45.8% and 21.9%, respectively. The distribution of dietary intakes, reported on the food records by the MEDAS-derived total MedDiet score, was in the expected direction, but no association was observed with cardiometabolic risk factors. The two administrations of the MEDAS produced similar mean total MedDiet scores (5.5 vs. 5.4, *p* = 0.706), which were correlated (*r* and ICC = 0.69, *p* < 0.001) and agreed fairly (*κ* = 0.38, 95% CI 0.24–0.52, *p* < 0.001; 95% limits of agreement −3.1, 3.2). The English version of the MEDAS has acceptable accuracy and reliability for assessing MedDiet adherence among individuals with a high cardiovascular risk, in the UK, and can be used to rank individuals according to MedDiet adherence in research and practice.

## 1. Introduction

Cardiovascular disease (CVD) is an important global public health problem associated with death and disability [1]. In the United Kingdom (UK), CVD is the second most common cause of death, resulting in 28% of all deaths [2]. In particular, coronary heart disease and stroke result in approximately 74,000 and 41,000 deaths in the UK every year, respectively [2]. In addition, approximately 25% of hospital admissions for CVD are in patients with type 2 diabetes (T2D) [3] and CVD accounts for 52% of mortality among these patients [4]. Approximately £6.8 billion is spent by the National Health Service (NHS) on treating CVD in England [2].

The Mediterranean diet (MedDiet), rich in olive oil, nuts, fruits and vegetables, whole grains and pulses, low-fat dairy, fish, moderate alcohol amounts and limited quantities of red meat and sweets, has been the factor most frequently cited to explain the low CVD incidence and mortality [5] as well as high life expectancy [6] observed in Mediterranean countries. A recent systematic review of prospective cohort studies showed that MedDiet adherence is associated with an 11% reduced risk of CVD incidence and/or mortality [7]. In addition, randomized controlled trials have shown that people at high risk of CVD who follow a MedDiet demonstrate favourable changes in cardiometabolic risk factors and decreased CVD incidence/mortality [7]. The Spanish PREDIMED study (‘Prevention with Diet Mediterranean’) examined the effect of the MedDiet, compared to the low-fat diet usually recommended for CVD prevention, on clinical CVD endpoints among Spanish high-CVD-risk patients [8] and found that the MedDiet reduced the incidence of CVD events by 29% [9]. Despite the recently recognized need to promote this dietary pattern to non-Mediterranean populations [10], the vast majority of evidence on the impact of the MedDiet on CVD is based on intervention trials in Mediterranean countries [11]. The PREDIMED results support the potential of promoting the MedDiet for the primary prevention of CVD in the UK, particularly when considering the high burden of CVD in this country [2].

To enable the assessment of the MedDiet in the UK and to measure change towards this dietary pattern in response to an intervention, it is important to identify an accurate and reliable instrument to assess MedDiet adherence. Dietary assessment via food frequency questionnaires (FFQs), food records and dietary recalls underpins the evidence base for the role of the MedDiet in health promotion, but is impractical for dietary counselling and provision of advice in nutrition interventions, since it increases burden for participants and practitioners alike [12]. In contrast, the use of short questionnaires evaluating MedDiet adherence can be a useful and simple means of examining the effect of this dietary pattern on health outcomes and aid in the provision of brief dietary feedback and nutrition education [13]. The PREDIMED study used a 14-item questionnaire (Mediterranean Diet Adherence Screener, MEDAS) for assessment and provision of immediate feedback to participants in the trial. This instrument was shown to be a valid tool for rapidly assessing and providing advice on MedDiet adherence among 7146 high-CVD-risk patients (Pearson’s *r*=0.52, *p* < 0.001; *κ* statistic = 0.43), when compared to an extensive, full-length FFQ [14]. The average MEDAS-derived MedDiet score estimate was 105% of the 136-item FFQ-derived MedDiet score estimate. Limits of agreement, according to the Bland–Altman method, ranged between 57 and 153%. Multiple linear regression analyses revealed that a higher MEDAS score related directly (*p* < 0.001) to high-density lipoprotein (HDL) cholesterol and inversely (*p* < 0.038) to body mass index, waist circumference, triglycerides (TG), the TG/HDL ratio, fasting glucose, and the total cholesterol/HDL ratio. The 10-year estimated coronary artery disease risk decreased as the 14-item PREDIMED score increased (*p* < 0.001) [14]. Subsequently, the validity of the German and English versions of the MEDAS were tested among 68 women at high risk for breast cancer in Germany [15] and 16 heart and lung transplant patients in England [16], respectively, demonstrating that it is a valid instrument for rapidly assessing MedDiet adherence. These three studies, however, validated the MEDAS against full-length FFQs, which, due to their similar design to the MEDAS, might result in similar measurement errors [17]. In contrast, comparing the MEDAS with food records might give a better indication of its accuracy in assessing MedDiet adherence, as the measurement errors of these methods are not correlated [17]. In addition, none of the aforementioned studies examined the test-retest reliability of the MEDAS, which is important for establishing its overall utility in research and practice. The aim of the present study was therefore to assess the validity (accuracy and reliability) of the English version of the MEDAS among a sample of patients with high CVD risk in the UK, prior to its use in an intervention to promote the MedDiet in the UK. We used food records to assess the concurrent validity of the MEDAS, to overcome the potential limitations of FFQ use as the comparison method.

## 2. Materials and Methods

### 2.1. Participants

During January 2015, all 34 general practices in the city of Bristol, UK, were invited by the Clinical Research Network (West of England) to facilitate the study, and ten expressed willingness. We recruited six general practices, purposively sampled to ensure variation in socioeconomic/ethnicity profiles of their populations, to undertake database searches, according to participant inclusion/exclusion criteria, and send an invitation letter to eligible patients (a detailed patient information sheet was enclosed with the invitation).

Similar to the PREDIMED study [8], eligible participants were community-dwelling high-risk individuals, aged 55–80 years (men) and 60–80 years (women), who met at least one of three inclusion criteria: (a) diagnosed T2D, (b) having ≥3 CVD risk factors (e.g., being overweight or having family history of CVD) or (c) having 10-year CVD risk ≥20%. The latter inclusion criterion was assessed using the QRISK2-2011 score [18] and was incorporated in the original PREDIMED inclusion criteria because it is commonly assessed in patients of the aforementioned ages who attend NHS Health Checks in the UK. Participant exclusion criteria included established CVD, medical conditions impairing participation in a nutrition intervention study, medical conditions limiting survival to <1 year, immunodeficiency, chronic alcoholism or excessive alcohol consumption (>80 g/day), body mass index >40 kg/m^2^, being institutionalized, lacking autonomy, unable to attend visits, participants whose high CVD risk had not been communicated to them by their doctor or inadequate understanding of verbal/written information given in English [8]. Participation was voluntary, and all participants provided written consent prior to data collection commencing. The study was conducted in accordance with the Declaration of Helsinki and the protocol was approved by the Health Research Authority, National Research (NRES) Committee South West—Central Bristol (reference 14/SW/1127).

### 2.2. Procedures

Data was collected between May and August 2015 at participants’ general practice premises by the same trained researcher, to minimize the potential for inter-interviewer reliability bias. Standard operating procedures were followed to ensure standardization of data collection among participants. The first appointment involved the collection of a non-fasting blood sample and anthropometric and blood pressure measurements, and the completion of an interviewer-administered demographic questionnaire and the interviewer-administered MEDAS. Participants were then provided with detailed verbal and written instructions to complete a 3-day estimated food record, which they were instructed to start completing approximately two weeks following their first appointment. A second appointment was arranged, approximately one month following the participant’s first appointment, where completed food records were returned and the MEDAS was administered again via an interview.

### 2.3. Cardiometabolic, Anthropometric and Blood Pressure Measurements

A non-fasting venous blood sample was collected in appropriate vacutainers by a researcher trained in venipuncture and sent to the Clinical Biochemistry Department of the Bristol Royal Infirmary on the day of collection, for total cholesterol, HDL-cholesterol, TG, non-HDL-cholesterol and glycosylated hemoglobin (HbA1c) analysis, using calibrated equipment. Low-density lipoprotein (LDL) cholesterol values were calculated using the Friedewald equation [19]. The ratios of total/HDL-cholesterol and TG/HDL-cholesterol were calculated.

Body weight was measured using a calibrated digital scale (Seca 899, Hamburg, Germany), to the nearest 0.1 kg, with the participant barefoot and in light clothing. Body height was measured using a wall-mounted calibrated stadiometer (Seca 225, Hamburg, Germany), to the nearest millimeter, with the participant barefoot. Body mass index (BMI) was calculated as weight divided by height, squared (kg/m^2^). Waist circumference was measured to the nearest millimeter, using a tape at a vertical plane, midway between the lower rib and iliac crest, and with the participant standing and gently breathing out. This measurement was obtained twice, and the mean was calculated.

Systolic (SBP) and diastolic (DBP) blood pressure measurements were obtained from the left arm with an electronic sphygmomanometer, with the participant sitting and after having rested quietly for five minutes. Blood pressure was obtained three times, with an interval of two minutes between measurements. The mean of the second and third measurements was calculated and presented in the current report.

### 2.4. Demographic Characteristics

Participants were asked to report their sex, date of birth, ethnicity [20], marital status [21], highest level of educational attainment [22], current employment status and current smoking status [8]. For ease of interpretation and analysis, categorical variables were collapsed where appropriate. ‘Ethnicity’ was dichotomized into ‘Caucasian’ and ‘other ethnicity’, and ‘marital status’ and ‘current employment status’ were collapsed into ‘single’, ‘married/cohabiting’, and ‘divorced/separated/widowed’, and ‘retired’, ‘employed’ and ‘unemployed’, respectively.

### 2.5. MEDAS

The MEDAS is a 14-item questionnaire requesting participants to report the habitual frequency of consumption or amount consumed of 12 main components of the MedDiet and two food habits related to the MedDiet [8,14]. Each of the 14 items is scored 1 or 0, depending on whether participants adhere to each MedDiet component or not. The MEDAS items and the criteria for scoring 1 point are shown in Table S1. If these conditions were not met, an item was assigned a score of 0. The resulting MEDAS-derived MedDiet score ranged from 0 to 14 [8,14]. Photographs of portion and serving sizes were used, as appropriate, to facilitate completion of the MEDAS [23].

### 2.6. Reference Instrument

A 3-day estimated food record [24] was used as the reference instrument to establish concurrent validity with the MEDAS. This method is considered acceptable for the assessment of usual dietary intake and is commonly used in dietary validation studies [17]. Participants were asked to record, in as much detail as possible, all foods and beverages consumed over three days (non-consecutive, including one weekend day), describe the amounts consumed in household measures (e.g., slices, cups, spoons) or provide weights, when known (e.g., packaged foods), report cooking methods and provide the recipes for any mixed dishes. Participants were provided with detailed verbal and written information on how to complete their food record and they were encouraged to contact a member of the research team if any questions arose during the procedure. Participants were requested to maintain their usual eating habits throughout the recording period.

Upon return, the food record was reviewed on site by the researcher for completeness with regards to portion sizes, cooking methods and description of foods, and any uncertainties were resolved with participants. Food records were coded by the same researcher, in order to minimize coding variability, and checked for accuracy by a second member of the research team (blinded with respect to the MEDAS score), using the Dietplan6 nutrition analysis software (Forestfield Software Ltd., Horsham, UK). Dietplan6 is based on the sixth edition of McCance and Widdowson’s Composition of Foods [25], updated with manufacturers’ data. A food item file was extracted from Dietplan6, listing the amount of all foods eaten at each eating occasion by each participant. All unique food items reported were exported to a list and allocated to a MEDAS relevant food group. The food item file was collapsed to compute average number of servings per day or week of each food group, daily frequency of use of different types of fat in cooking or types of meat and weekly frequency of use of sofrito, so that whether participants met the criteria for each component of the MEDAS could be assessed (Table S1). The total MedDiet score was computed for each participant from food record data based on the number of 14 criteria met.

### 2.7. Statistical Analyses

We estimated that a sample size of 100 participants would have 80% power to detect a correlation in the total MedDiet score between the MEDAS and the 3-day food record of at least 0.3 (alpha of 5%, power of 80%). This would give 95% confidence intervals (CIs) for limits of agreement of approximately ±0.34 standard deviations [14]. All analyses were performed using the Statistical Package for the Social Sciences (IBM SPSS Statistics, SPSS, Chicago, IL, USA). Descriptive statistics (means, standard deviations, *n* and percentages) were used to report demographic and clinical characteristics of participants.

#### 2.7.1. Concurrent Validity

The mean total MedDiet score was calculated from the MEDAS and the 3-day estimated food record. Pearson’s *r* correlation coefficient and intra-class correlation coefficients (ICC, two-way mixed effects model with average measures) were used to assess the relative agreement between the two dietary assessment methods. The mean MedDiet score, derived from the MEDAS and the food record, was categorized into the same three equal groups (0–3, 3.1–4.7, 4.8–14). Participants’ cross-classification and the percentage of participants classified into the same, adjacent and opposite categories were determined and agreement within categories between the two dietary assessment methods was then estimated using the kappa (*κ*) statistic. In validation studies of dietary assessment instruments, *κ* values from 0 to 0.20 indicate poor agreement, 0.21 to 0.40 fair agreement, 0.41 to 0.60 moderate agreement, 0.61 to 0.80 good agreement and 0.81 to 1 very good agreement [26]. Bland and Altman plots with 95% limits of agreement (mean difference between the MedDiet score derived from the MEDAS and food records ± 1.96 × SD of the mean difference) determined absolute agreement in the total MedDiet score between the two methods. These plots display the difference between estimates of the same score (MedDiet score) from two methods against the average score from both methods and assess the relationship between the measurement error and the true value, with a mean difference of 0 indicating complete agreement between the methods [27,28]. Agreement between the individual items of the MEDAS and those derived from the food records was also assessed, using the *κ* statistic, to understand if concordance in the total MedDiet score was driven by individual score components.

#### 2.7.2. Predictive Validity

Linear regression models were fitted to examine the association between the total MEDAS-derived MedDiet score with cardiometabolic, anthropometric and blood pressure variables. General ordinary least-squares linear modeling was used to estimate energy-adjusted food and nutrient intakes, derived from the food records, according to tertile distribution of the MEDAS-derived MedDiet score (entered as a continuous variable in the model). The linear trend was assessed by polynomial contrasts and a post hoc Bonferroni correction was used for multiple comparisons.

#### 2.7.3. Test-Retest Reliability

The mean total MedDiet score was calculated from the first and second administrations of the MEDAS. Pearson’s *r* correlation coefficient and ICC (two-way mixed effects model with average measures) were used to assess relative agreement between the two repeated MEDAS administrations. The mean MedDiet score was categorized into three equal groups, separately for each MEDAS administration (MedDiet score groups for MEDAS’ first administration: 0–4.3, 4.4–6, 6.1–14; MedDiet score groups for MEDAS’ second administration: 0–5, 5.1–6, 6.1–14).

Participant cross-classification and the percentage of them classified into the same, adjacent and opposite categories were established, and agreement within categories between the first and second MEDAS administration was then estimated using the kappa (*κ*) statistic and Bland and Altman plots with 95% limits of agreement.

## 3. Results

### 3.1. Participant Characteristics

Ninety-nine patients at high CVD risk were recruited from six general practices in Bristol (mean age 68.3 years, mean BMI 28.3 kg/m^2^). Table 1 presents the demographic characteristics and cardiometabolic, anthropometric and blood pressure measurements of participants. The majority of participants were women (66%), Caucasian (93%), married/cohabiting (55%), retired (76%) and overweight/obese (76%).

### 3.2. Concurrent Validity

Ninety-six participants completed the MEDAS at their first appointment and returned a food record. Compared to food records, the MEDAS estimated a higher mean total MedDiet score by 1.47 points (5.47 ± 2.09 vs. 4.00 ± 1.47, 95% CI 1.09–1.85). There was strong evidence of a moderate correlation between the MEDAS-derived MedDiet score and the food record MedDiet score (*r* = 0.50, *p* < 0.001; ICC = 0.53, 95% CI 0.07–0.74, *p* < 0.001). The proportion of participants classified into the same category of the total MedDiet score by the MEDAS and the food records was 45.8%, whereas gross misclassification (classification into opposite categories) was 21.9%. The *κ* statistic was 0.19 (95% CI 0.07–0.31, *p* = 0.002), indicating borderline fair agreement between the two dietary assessment methods [26].

Table 2 demonstrates the proportion of participants who met the criteria for achieving each of the 14 points of the score corresponding to each component of the MedDiet by the MEDAS and by the food records. A higher proportion of participants achieved a score of 1 via the MEDAS, compared to the food records, for fruit (36.5 vs. 25.0%, *p* < 0.001), red meat (78.1 vs. 71.9%, *p* = 0.002), wine (30.2 vs. 27.1%, *p* < 0.001), nuts (33.3 vs. 21.9%, *p* = 0.017) and sofrito (10.4 vs. 6.3%, *p* = 0.014). Concordance was highest for wine (*κ* = 0.72, *p* < 0.001), whereas total olive oil consumed, and pulses had no agreement. Overall, poor, fair, moderate and good concordance was found for 57.1, 28.6, 7.1 and 7.1% of the components of the MedDiet score, respectively.

**Figure 1:**
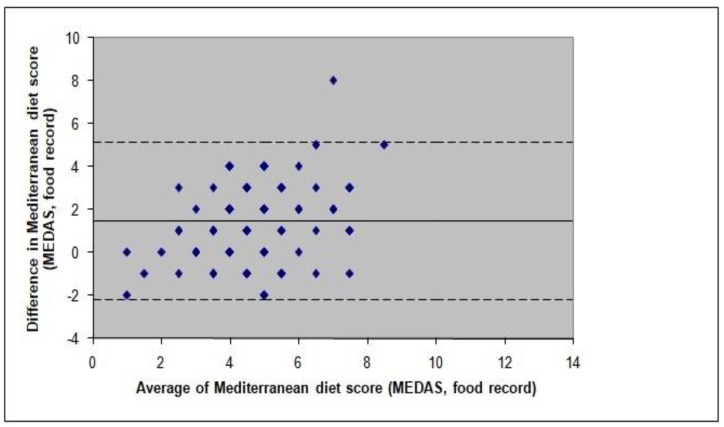
Bland and Altman plot and limits of agreement for the total Mediterranean diet score as estimated by the MEDAS and the 3-day estimated food record. The unbroken line indicates the mean bias, and the dashed lines indicate the limits of agreement. MEDAS, Mediterranean Diet Adherence Screener.

The Bland–Altman plot for the total MedDiet score from the two dietary assessment methods is illustrated in Figure 1. The calculation of the mean difference between the two methods (1.47 ± 1.86, 95% limits of agreement −2.2, 5.1) confirmed that the total MedDiet score calculated by the MEDAS was slightly higher, compared to that calculated from the food records.

### 3.3. Predictive Validity

Linear regression analyses found no evidence against the null hypothesis of no associations between the total MedDiet score, derived by the MEDAS, and cardiometabolic, and anthropometric and blood pressure variables (Table 3).

The distribution of food and nutrient intakes calculated from the food records by tertiles of the MEDAS-derived MedDiet score were generally in the expected direction, with strong levels of evidence observed for olive oil, vegetable, red meat and sugar-sweetened beverage consumption as well as intake of carbohydrates, total fat, monounsaturated and polyunsaturated fat and vitamin E (Table 4).

### 3.4. Test-Retest Reliability 

**Figure 2:**
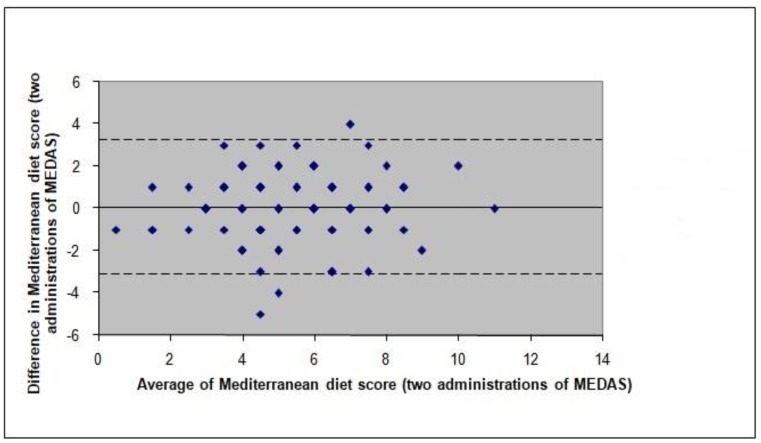
Bland and Altman plot and limits of agreement for the total Mediterranean diet score as estimated by the MEDAS’ two administrations. The unbroken line indicates the mean bias and the dashed lines indicate the limits of agreement. MEDAS, Mediterranean Diet Adherence Screener.

Ninety-six participants completed the MEDAS twice for reliability testing. The two administrations of the MEDAS produced a similar mean total MedDiet score (5.5 ± 2.1 vs. 5.4 ± 2.0, *p* = 0.706) and relative agreement was good (*r* = 0.69, *p* < 0.001; ICC = 0.69, 95% CI 0.571–0.783, *p* < 0.001), indicating good reliability [12,29]. The proportion of participants classified into the same category of the total MedDiet score distribution by the two MEDAS administrations was 58.3%, whereas gross misclassification (classification into opposite categories) was only 8.3%. The *κ* was 0.38 (95% CI 0.24–0.52, *p* < 0.001), indicating fair agreement between the two MEDAS administrations [26]. The calculation of the mean MedDiet score difference between the two MEDAS administrations (0.06 ± 1.62, 95% limits of agreement −3.1, 3.2) confirmed that the two administrations of the MEDAS resulted in similar total MedDiet scores (i.e., almost null bias) (Figure 2).

## 4. Discussion

The aim of the current study was to examine the validity of the English version of the MEDAS [14] in assessing adherence to the MedDiet among a sample of high-CVD-risk individuals in the UK. This work extends earlier research that assessed the relative validity of the English version of the MEDAS in a small sample of adults within tertiary care [16], by recruiting a larger sample of adults who were CVD free, using detailed food records as the criterion measure and examining the MEDAS’ test-retest reliability. We showed that the English version of the MEDAS provided a borderline fair estimate of MedDiet adherence, when compared to the reference instrument of 3-day estimated food records and similar rankings of participants on the basis of their MedDiet score. Test-retest reliability indicated that the English MEDAS is a reliable instrument to assess MedDiet adherence, with good stability over time.

The moderate relative agreement between the MEDAS- and food record-derived MedDiet scores observed in our study is comparable to the first study assessing the accuracy of the MEDAS among Spanish older adults at high risk of CVD (Pearson’s *r* = 0.52, *p* < 0.001; ICC = 0.51, *p* < 0.001) [14]. This suggests that the potential of the MEDAS to assess adherence to the MedDiet is similar in both Mediterranean and non-Mediterranean countries. In contrast, relative agreement between the MedDiet score derived from the MEDAS and the MedDiet score derived from an FFQ was higher among heart and lung transplant patients in the UK (Pearson’s *r* = 0.72, *p* = 0.002; ICC = 0.64, 95% CI 0.31–0.85) [16]. Further, the absolute agreement between the MEDAS- and food record-derived MedDiet scores in our study was lower than the moderate agreement (*κ* = 0.43) demonstrated between the MEDAS and an FFQ in Spain [14]. We acknowledge, however, that our sample size is considerably smaller than the >7000 participants assessed in the Spanish study and we may have suboptimal statistical power for some comparisons. With regard to individual dietary components of the MedDiet score, we found that concordance between the MEDAS and the food records was poor for 57.1% of components, compared to 21.4% and 28.6% in the Spanish [14] and German [15] validation studies of the MEDAS against an FFQ, respectively. Nevertheless, the present study showed that exact agreement (45.8%) within categories of the total MedDiet score between the dietary assessment methods under comparison was comparable to the Spanish study (47.9%) [14]. Classification into opposite categories of the MEDAS and the reference instrument was higher in the current, compared to the Spanish, study (21.9 vs. 8.6%) [14], which might have resulted from the small sample size in our study. Overall, our findings suggest that the MEDAS provides reasonable estimates to assign the same absolute MedDiet score ratings as the food records [29].

It should be noted, however, that comparisons of earlier studies with the current study should be undertaken with caution, due to the different reference instruments utilized to assess the accuracy of the MEDAS. For example, the reference dietary assessment method in all earlier MEDAS validation studies was a full-length validated FFQ, assessing dietary habits over the previous 12 months [14,15,16]. Although FFQs have their own limitations [30], the reference period of one year in these earlier studies might have captured habitual dietary habits, as well as food items affected by seasonal variation, more accurately than the 3-day food record used in the current study. Also, it has been suggested that when instruments assessing habitual diet, such as the MEDAS, are validated against food records, a certain degree of disagreement is to be expected, due to within-subject variations that naturally occur over the shorter reference period of the food record [17]. Further, because the MEDAS has a similar design to an FFQ [14], comparing it against the latter will most likely overestimate its accuracy, due to similarities in measurement errors [17]. Specifically regarding the earlier research in England, it might also be that the specific health status of participants (individuals with already established disease) [16] encouraged them to give more accurate responses in both dietary assessment methods [17].

Differences in the intakes of the MEDAS food components between the studies might also account for the observed differences in absolute agreement. For example, only 1% of participants in our sample met the criterion for olive oil intake by both the MEDAS and food records, compared to 70.9% and 63.9% of Spanish adults meeting this criterion by the MEDAS and FFQ, respectively [14]. These higher levels of olive oil intake in the Spanish study might have given greater power to observe strong evidence of agreement and might explain the poor agreement in some food components in our study. Nevertheless, we consider that testing the MEDAS’ accuracy against food records is a strength of our study. Although not free of error in assessing habitual dietary intake, food records have been suggested to be the preferred method of choice when validating instruments such as the MEDAS, as they tend to minimize errors related to recall and perceptions of portion sizes and, unlike FFQs, the measurement errors of the MEDAS and food records are not correlated [17]. In addition, administering the MEDAS prior to, and independently from, the reference instrument, is a strength of our study, as this sequence of administration minimises the risk of the training effect on diet recording (i.e., providing answers according to what was recorded on the reference instrument), and it is the procedure recommended for dietary validation studies [17]. However, the possibility remains for reactivity, i.e., that participants eat differently during the 3 days of food recording because of completing the MEDAS questionnaire previously. To our knowledge, there is no study randomizing the order of completion of FFQ and food records to understand the possible effect of prior exposure to a brief tool. In general, reactivity within food records typically accounts for 10% of energy intake [31,32] and is observed across all food groups [33], suggesting that no specific effects on the overall Mediterranean diet score would be observed.

In the current study, participants who were in the highest tertile of MedDiet adherence, as indicated by the MEDAS-derived MedDiet score, had higher intakes of olive oil, vegetables, total, monounsaturated and polyunsaturated fat and vitamin E, and lower intakes of red/processed meat and sugar-sweetened beverages, compared to participants in the lowest MedDiet adherence tertile. These findings are in agreement with the original validation study of the MEDAS in older Spanish adults [14]. The evidence for the association between the MEDAS-derived MedDiet score and other foods and nutrients calculated from the food records was weak. However, all distributions were in the expected direction, suggesting that, in this respect, the English MEDAS provides acceptable levels of predictive validity and that it reflects the intake of specific foods and nutrients typical of the traditional MedDiet [34,35]. Despite the evidence for the beneficial role of the MedDiet on cardiometabolic risk factors [36,37,38,39], no associations were observed between the total MEDAS-derived MedDiet score and cardiometabolic, anthropometric and blood pressure measurements, probably because of suboptimal statistical power in our study. This is in contrast to the substantially larger Spanish study, which found that the MEDAS-derived MedDiet score was inversely associated with BMI, waist circumference, TG, total/HDL-cholesterol and TG/HDL-cholesterol ratios, and was positively associated with HDL-cholesterol [14]. It might be that specific aspects of the UK diet (i.e., more processed foods, ready meals, savoury snacks and pastries, etc.) [24], which are not captured by the MEDAS, outweighed the benefits of adhering to some MedDiet components. It is also noteworthy that the mean MEDAS-derived MedDiet score in the current study was lower than the one observed in Spanish adults (5.47 vs. 8.68) [14]. This lack of variation in the MedDiet score, in addition to the smaller sample size compared to the Spanish study, even if acceptable for a dietary validation study [17], contributed to our study not having adequate power to detect associations between the MEDAS-derived MedDiet score and these variables. Considering the benefits of adhering to the MedDiet on CVD prevention, as demonstrated by the PREDIMED study [9], the low overall adherence to the MedDiet among participants in the current study emphasises the need for the development of strategies to help individuals at high CVD risk in the UK to adopt this health-promoting dietary pattern.

Limitations of the study include the self-selection of older adults at high risk of CVD as participants, who might already have made favourable changes to their diet, due to their knowledge of their risk. It is noteworthy, however, that participants had low adherence to the MedDiet, as indicated by both the MEDAS- and food record-derived MedDiet scores, which suggests that further dietary changes could be made to improve health. Our findings might not be generalisable to younger, healthy adults, and further assessment among a more ethnically and socio-economically diverse sample would establish the English MEDAS’ wider utility potential. There is no reason to believe that dietary reporting would vary in other populations; however, further validation studies would confirm this. Finally, the design of our study did not allow the assessment of the MEDAS’ ability to detect changes in MedDiet adherence and therefore, its potential utility in the evaluation of nutrition interventions aiming to promote the MedDiet. Nevertheless, the PREDIMED study demonstrated that the MEDAS can detect meaningful increases in MedDiet adherence following intervention [40], so there is good reason to believe the same would be true of the MEDAS’ English version. Also, the observed stability of the MEDAS, when examining its test-retest reliability, indicates that any observed differences in the MedDiet score derived by two consecutive administrations of the MEDAS would likely be the result of an intervention effect, instead of secular instability of the instrument [41]. However, evidence from an intervention study utilising the English version of the MEDAS to assess changes in MedDiet adherence, compared to a reference dietary assessment method, would help to confirm this.

## 5. Conclusions

Despite the aforementioned limitations, in this sample of adults at high risk of CVD in a non-Mediterranean country, the English version of the MEDAS provided an acceptable estimate of adherence to the MedDiet against the reference instrument of estimated food records, as well as reasonable rankings according to the derived MedDiet score. Further, the MEDAS had good test-retest reliability, indicating that it is a reliable instrument for assessing MedDiet adherence over time. Considering the need to identify valid dietary assessment instruments that reduce burden for research participants and researchers, as well as patients and practitioners [12], the English MEDAS can therefore be used to rapidly assess adherence to the MedDiet and provide feedback to individuals wishing to make changes towards this dietary pattern in research and clinical practice.

## Figures and Tables

**Table 1:** Demographic characteristics and cardiometabolic, anthropometric and blood pressure measurements of participants (*n* = 99).

Participant Characteristics	*n*	%
Sex		
Males	34	34.3
Females	65	65.7
Ethnic background		
Caucasian	92	92.9
Other	7	7.1
Marital status		
Single	14	14.1
Married/cohabiting	54	54.6
Divorced/separated/widowed	31	31.3
Education		
No qualifications	31	31.3
GCE ‘O’ levels, CSE, GCSE	21	21.2
GCE ‘A’ level or equivalent	11	11.1
Further education (e.g., HNC, HND)	4	4.1
Degree or equivalent	21	21.2
Postgraduate degree	11	11.1
Occupation		
Retired	75	75.8
Employed	23	23.2
Unemployed	1	1
Current smokers ^1^	15	15.1
Body weight status		
Underweight	2	2
Normal weight	21	21.4
Overweight	42	42.9
Obese	33	33.7
Age, years ^2^	68.3 ± 6.0 (56–80)	
Body mass index, kg/m^2^ (*n* = 98) ^3^	28.3 ± 4.4	
Waist circumference, cm (*n* = 99) ^3^	99.3 ± 11.9	
Systolic blood pressure, mmHg (*n* = 95) ^3^	133.8 ±17.1	
Diastolic blood pressure, mmHg (*n* = 95) ^3^	79.5 ± 9.5	
Total cholesterol, mmol/L (*n* = 87) ^3^	5.5 ± 1.2	
LDL-cholesterol, mmol/L (*n* = 86) ^3^	3.2 ± 1.3	
HDL-cholesterol, mmol/L (*n* = 87) ^3^	1.6 ± 0.5	
Non-HDL-cholesterol, mmol/L (*n* = 87) ^3^	3.9 ± 1.1	
Triglycerides, mmol/L (*n* = 87) ^3^	1.7 ± 0.9	
Total:HDL-cholesterol ratio (*n* = 87) ^3^	3.7 ± 1.2	
Triglyceride:HDL-cholesterol ratio (*n* = 87) ^3^	1.2 ± 0.9	
Glycosylated haemoglobin (HbA1c), % (*n* = 54) ^3^	6.0± 3.1	

GCE, general certificate of education; CSE, certificate of secondary education; GCSE, general certificate of secondary education; HNC, higher national certificate; HND, higher national diploma; LDL, low-density lipoprotein; HDL, high-density lipoprotein.^1^ Current and ex-smokers (up to 1 year).^2^ Values are mean ± standard deviation (range).^3^ Values are mean ± standard deviation.

**Table 2:** Percentage of participants achieving the corresponding point for each of the 14 components of the Mediterranean diet score in the MEDAS (first administration) and food records (*n* and %), and agreement between the two dietary assessment methods (*n* = 96).

	MEDAS	3-Day Food Record	*κ* (95% CIs)	*p*-Value
Olive oil for cooking	50 (52.1)	38 (39.6)	0.09 (−0.12, 0.28)	0.356
Total olive oil consumed	1 (1.0)	1 (1.0)	−0.01 (−0.03, 0.00)	0.918
Vegetables	10 (10.4)	5 (5.2)	0.21 (−0.07, 0.52)	0.026
Fruit	35 (36.5)	24 (25.0)	0.49 (0.29, 0.66)	<0.001
Red and processed meat	75 (78.1)	69 (71.9)	0.34 (0.11, 0.54)	0.001
Butter, margarine, cream	58 (60.4)	51 (53.1)	0.18 (−0.02, 0.37)	0.080
Sugar sweetened beverages	69 (71.9)	76 (79.2)	0.19 (−0.04, 0.40)	0.059
Wine	29 (30.2)	26 (27.1)	0.72 (0.55, 0.86)	<0.001
Pulses	12 (12.5)	12 (12.5)	−0.05 (−0.17, 0.15)	0.641
Fish and seafood	49 (51.0)	39 (40.6)	0.17 (−0.02, 0.35)	0.089
Sweets and pastries	22 (22.9)	11 (11.5)	0.03 (−0.15, 0.24)	0.715
Nuts	32 (33.3)	21 (21.9)	0.26 (0.05, 0.44)	0.009
Preference for white over red meat	73 (76.0)	5 (5.2)	0.03 (0.31, 0.33)	0.197
Sofrito	10 (10.4)	6 (6.3)	0.32 (0.01, 0.02)	0.015

95% CIs, 95% confidence intervals; MEDAS, Mediterranean Diet Adherence Screener.

**Table 3:** Associations between the total Mediterranean diet score derived by the MEDAS (first administration) and cardiometabolic, anthropometric and blood pressure variables.

Dependent Variable	Unstandardised Regression Coefficient ^a^	95% CIs	*p*-Value
Body mass index, kg/m^2^	−0.044	−0.492, 0.404	0.847
Waist circumference, cm	−0.102	−1.263, 1.060	0.862
Systolic blood pressure, mmHg	0.229	−1.742, 1.713	0.799
Diastolic blood pressure, mmHg	0.232	−0.781, 1.246	0.650
Total cholesterol, mmol/L	−0.024	−0.151, 0.103	0.708
LDL-cholesterol ^b^, mmol/L	−0.013	−0.032, 0.006	0.181
HDL-cholesterol, mmol/L	−0.012	−0.062, 0.038	0.632
Non-HDL-cholesterol, mmol/L	−0.012	−0.129, 0.105	0.839
Triglycerides ^b^, mmol/L	0.010	−0.013, 0.034	0.380
Total:HDL-cholesterol ratio ^b^	0.002	−0.011, 0.016	0.731
Triglyceride:HDL-cholesterol ratio ^b^	0.015	−0.016, 0.047	0.340
HbA1c ^b^, %	0.006	−0.008, 0.020	0.400

95% CIs, 95% confidence intervals; HbA1c, glycosylated hemoglobin; HDL, high-density lipoprotein; LDL, low-density lipoprotein; MEDAS, Mediterranean Diet Adherence Screener.^a^ Linear regression analysis, adjusted for age, sex, smoking status, educational status, and marital status—the β coefficient represents a one-unit change in dependent variables for each one point increase in the MEDAS-derived Mediterranean diet score.^b^ Log transformed.

**Table 4:** Energy-adjusted food and nutrient intake recorded on the food records according to tertile distribution of the Mediterranean diet score derived by the MEDAS ^a^.

	1st Tertile (score = 0–4, *n* = 32)	2nd Tertile (score = 5–6, *n* = 34)	3rd Tertile (score = 7–14, *n* = 30)	*p*-Linear Trend ^b^
Foods				
Olive oil (g/4.18 MJ)	0.6 (0.1, 1.1)	2.2 (1.1, 3.5)	4.3 (2.5, 6.7)	0.001
Vegetables (g/4.18 MJ)	78.0 (57.6, 100.4)	130.7 (103.6, 160.9)	170.3 (132.3, 214.6)	0.001
Fruits (g/4.18 MJ)	87.5 (57.2, 128.9)	103.6 (74.5, 145.0)	154.1 (110.0, 206.2)	0.085
Red and processed meat (g/4.18 MJ)	70.1 (50.0, 90.1)	39.8 (29.9, 50.5)	32.7 (21.6, 44.3)	0.001
Butter and animal fats (g/4.18 MJ)	11.0 (7.5, 14.7)	10.3 (7.1, 13.8)	7.0 (4.7, 9.6)	0.101
Sugar-sweetened beverages (g/4.18 MJ)	77.8 (39.5, 119.4)	18.3 (8.6, 30.7)	9.1 (0.2, 23.1)	0.001
Wine (g/4.18 MJ)	43.0 (16.9, 78.6)	52.6 (28.7, 80.0)	49.5 (24.7, 77.2)	0.967
Pulses (g/4.18 MJ)	22.8 (14.8, 32.5)	15.9 (8.6, 25.7)	32.3 (9.5, 66.2)	0.410
Nuts (g/4.18 MJ)	2.4 (0.8, 4.4)	3.6 (1.6, 6.3)	4.9 (2.4, 7.6)	0.103
Fish (g/4.18 MJ)	34.0 (21.3, 49.1)	28.8 (21.0, 37.0)	49.8 (36.9, 64.0)	0.094
Nutrients				
Protein (% of energy intake)	16.4 (15.2, 17.6)	17.2 (15.6, 19.1)	17.9 (16.6, 19.2)	0.086
Carbohydrates (% of energy intake)	47.8 (44.9, 50.7)	42.3 (39.6, 44.8)	41.8 (38.9, 44.7)	0.007
Total fat (% of energy intake)	33.7 (31.6, 35.8)	38.1 (35.6, 40.8)	37.9 (35.3, 40.9)	0.021
Saturated fat (% of energy intake)	12.8 (11.7, 13.9)	13.5 (12.1, 15.2)	11.6 (10.3, 13.2)	0.364
Trans fat (% of energy intake)	0.6 (0.5, 0.7)	0.7 (0.5, 0.8)	0.5 (0.4, 0.6)	0.511
Monounsaturated fat (% of energy intake)	12.1 (11.2, 13.0)	14.2 (13.0, 15.6)	15.0 (13.8, 16.3)	0.002
Polyunsaturated fat (% of energy intake)	4.6 (4.0, 5.3)	4.5 (3.9, 5.2)	5.7 (5.0, 6.5)	0.022
Dietary fibre (g/4.18 MJ)	11.0 (9.7, 12.6)	11.7 (10.5, 13.1)	13.5 (12.2, 15.3)	0.099
Dietary cholesterol (mg/4.18 MJ)	143.6 (123.4, 162.9)	166.0 (138.6, 193.9)	157.0 (128.1, 188.1)	0.279
Carotenes (ug/4.18 MJ)	1396.6 (985.7, 1854.9)	2488.3 (1747.6, 3299.4)	2275.5 (1544.1, 3165.6)	0.179
Vitamin E (mg/4.18 MJ)	4.8 (4.2, 5.4)	5.1 (4.6, 5.6)	6.3 (5.7, 6.8)	0.001
Folate (ug/4.18 MJ)	141.4 (126.1, 159.1)	154.6 (138.4, 171.0)	164.7 (151.0, 179.9)	0.107
Vitamin C (mg/4.18 MJ)	69.5 (49.9, 90.3)	71.6 (56.7, 89.0)	81.8 (67.1, 98.4)	0.711

MEDAS, Mediterranean Diet Adherence Screener.^a^ Values are means or percentages (95% CIs).^b^ General linear modeling, adjusted for age, sex, smoking status, educational status and marital status.

## Supplementary Materials

The following are available online at http://www.mdpi.com/2072-6643/10/2/138/s1, Table S1: Mediterranean Diet Adherence Screener (MEDAS) items and allocation of food intake data from food records into its food groups.
