# Supplementary material for: Validation of the English Version of the 14-Item Mediterranean Diet Adherence Screener of the PREDIMED Study, in People at High Cardiovascular Risk in the UK
Source: Nutrients. 2018 Jan 28;10(2):138. doi: 10.3390/nu10020138 (PMC5852714; doi:10.3390/nu10020138)
Supplement: Supplementary file 1 [file nutrients-10-00138-s001.pdf]

Supplementary Materials I

Table S1 Mediterranean Diet Adherence Screener (MEDAS) items and allocation of food intake data from food records into its food groups

MEDAS item	Criteria for obtaining 1 point	Food (and sub-food) groups created from food record data to derive the MEDAS food groups	Data from food records used to calculate MEDAS food groups
1. Do you use olive oil as the principal source of fat for cooking?	Yes	Olive oil:<br>2F. Vegetables cooked with olive oil<br>2J. Mixed vegetable dishes cooked with olive oil<br>4B. Red meat cooked with olive oil<br>4F. Mixed red meat dishes cooked with olive oil<br>8B. Pulses cooked with olive oil<br>8F. Mixed pulse dishes cooked with olive oil<br>9B. Fish cooked with olive oil<br>9F. Mixed fish dishes cooked with olive oil<br>9J. Shellfish/seafood cooked with olive oil<br>9N. Mixed shellfish/seafood dishes cooked with olive oil<br>12B. Poultry and rabbit dishes cooked with olive oil<br>14B. Miscellaneous foods cooked with olive oil<br><br>Butter, margarine, cream:<br>2G. Vegetables cooked with butter, margarine, cream<br>2K. Mixed vegetable dishes cooked with butter, margarine, cream	Average olive oil per 100g of total dish = (olive oil quantity/100) * 13.5g<br>Average butter/margarine/cream per 100g of total dish = (butter/margarine/cream quantity/100) * 12g<br>Average other fats per 100g of total dish (other fats quantity/100)* 13.5g<br><br>Total olive oil (g) = ‘2F’ (g) + ‘2J’ (g) + ‘4B’ (g) + ‘4F’ (g) + ‘8B’ (g) + ‘8F’ (g) + ‘9B’ (g) + ‘9F’ (g) + ‘9J’ (g) + ‘9N’ (g) + ‘12B (g) + ‘14B’ (g)<br><br>Total butter, margarine, cream (g) = ‘2G’ (g) + ‘2K’ (g) + ‘4C’ (g) + ‘4G’ (g) + ‘8C’ (g) + ‘8G’ (g) + ‘9C’ (g) + ‘9G’ (g) + ‘9K’ (g) + ‘9O’ (g) + ‘12C (g) + ‘14C’ (g)<br><br>Total other fats (g) = ‘2H (g) + ‘2L’ (g) + ‘4D’ (g) + ‘4H’ (g) + ‘8D’ (g) + ‘8H’ (g) + ‘9D’ (g) + ‘9H’ (g) +

	4C. Red meat cooked with butter, margarine, cream<br>4G. Mixed red meat dishes cooked with butter, margarine, cream<br>8C. Pulses cooked with butter, margarine, cream<br>8G. Mixed pulse dishes cooked with butter, margarine, cream<br>9C. Fish cooked with butter, margarine, cream<br>9G. Mixed fish dishes cooked with butter, margarine, cream<br>9K. Shellfish/seafood cooked with butter, margarine, cream<br>9O. Mixed shellfish/seafood dishes cooked with butter, margarine, cream<br>12C. Poultry and rabbit dishes cooked with butter, margarine, cream<br>14C. Miscellaneous foods cooked with butter, margarine, cream<br><br>Other fats:<br>2H. Vegetables cooked with other fats<br>2L. Mixed vegetable dishes cooked with other fats<br>4D. Red meat cooked with other fats<br>4H. Mixed red meat dishes cooked with other fats<br>8D. Pulses cooked with other fats<br>8H. Mixed pulse dishes cooked with other fats<br>9D. Fish cooked with other fats<br>9H. Mixed fish dishes cooked with other fats	‘9L’ (g) + ‘9P’ (g) + ‘12D (g) + ‘14D’ (g)<br><br>1 point: Total olive oil (g) > [Total butter, margarine, cream (g) + Total other fats (g)]

		9L. Shellfish/seafood cooked with other fats<br>9P. Mixed shellfish/seafood dishes cooked with other fats<br>12D. Poultry and rabbit dishes cooked with other fats<br>14D. Miscellaneous foods cooked with other fats	
2. How much olive oil do you consume per day (including that used in frying, meals eaten away from home, salads, etc.)? *(1 tablespoon = 13.5g)*	4 or more tablespoons	1. Olive oil (raw, e.g. added to salad)<br>2B. Salad and raw vegetables with olive oil<br>2F. Vegetables cooked with olive oil<br>2J. Mixed vegetable dishes cooked with olive oil<br>4B. Red meat cooked with olive oil<br>4F. Mixed red meat dishes cooked with olive oil<br>8B. Pulses cooked with olive oil<br>8F. Mixed pulse dishes cooked with olive oil<br>9B. Fish cooked with olive oil<br>9F. Mixed fish dishes cooked with olive oil<br>9J. Shellfish/seafood cooked with olive oil<br>9N. Mixed shellfish/seafood dishes cooked with olive oil<br>12B. Poultry and rabbit dishes cooked with olive oil<br>14B. Miscellaneous foods cooked with olive oil<br>All foods/meals prepared or cooked with olive oil	Total olive oil (g) = ‘1’ (g) + ‘2B (g) + ‘2F’ (g) + ‘2J’ (g) + ‘4B’ (g) + ‘4F’ (g) + ‘8B’ (g) + ‘8F’ (g) + ‘9B’ (g) + ‘9F’ (g) + ‘9J’ (g) + ‘9N’ (g) + ‘12B (g) + ‘14B’ (g)<br>Total olive oil (servings) = Total olive oil (g)/13.5<br><br>1 point: Total olive oil (servings) ≥4
3. How many servings of vegetables do you consume per day? *(1 serving = 200g - side dishes count as ½ a serving, not including potatoes or sweetcorn)*	2 or more (at least 1 portion raw or as salad)	2A. Salad and raw vegetables with no fat<br>2B. Salad and raw vegetables with olive oil<br>2C. Salad and raw vegetables with butter, margarine, cream<br>2D. Salad and raw vegetables with other fats	All vegetables (raw/salad and cooked):<br>Total vegetables (g) = ‘2A’ (g) + ‘2B’ (g) + ‘2C’ (g) + ‘2D’ (g) + ‘2E’ (g) + ‘2F’ (g) + ‘2G’ (g) + ‘2H’ (g) + ‘2I’ (g) + ‘2J’ (g) + ‘2K’ (g) + ‘2L’ (g)

		2E. Vegetables cooked with no fat<br>2F. Vegetables cooked with olive oil<br>2G. Vegetables cooked with butter, margarine, cream<br>2H. Vegetables cooked with other fats<br>2I. Mixed vegetable dishes cooked with no fat<br>2J. Mixed vegetable dishes cooked with olive oil<br>2K. Mixed vegetable dishes cooked with butter, margarine, cream<br>2L. Mixed vegetable dishes cooked with other fats<br>All vegetables (excluding potatoes and sweetcorn), including home-prepared, retail, in ready meals and canned varieties	Total vegetables (servings) = Total vegetables (g)/200<br><br>Raw vegetables/salad:<br>Total raw vegetables (g) = '2A' (g) + '2B' (g) + '2C' (g) + '2D' (g)<br>Total raw vegetables (servings) = Total raw vegetables (g)/200<br><br>1 point: Total vegetables (servings) ≥2 and total raw vegetables (servings) ≥1
4. How many pieces of fruit (including fresh-squeezed fruit juice) do you consume per day? *(not including frozen or dried fruit)*	3 or more	3A. Raw fresh fruit (not canned)<br>3B. Fresh fruit juice (freshly squeezed)<br>3C. Freshly cooked fruits	Total fresh fruit (g) = '3A' (g) + '3C' (g)<br>Total fresh fruit (servings) = Total fresh fruit (g)/80<br><br>Total fresh fruit juice (ml) = '3B' (ml)<br>Total fresh fruit juice (servings) = Total fresh fruit juice (ml)/100<br><br>Total fruit (servings) = Total fresh fruit (servings) + Total fresh fruit juice (servings)<br><br>1 point: Total fruit (servings) ≥3
5. How many servings of red meat, hamburger, or meat	Less than 1	4A. Red meat cooked with no fat	Total red meat (g) = '4A' (g) + '4B' (g) + '4C' (g) +

products (ham, sausage, etc.) do you consume per day?<br>*(1 serving = 100-150g)*		4B. Red meat cooked with olive oil<br>4C. Red meat cooked with butter, margarine, cream<br>4D. Red meat cooked with other fats<br>4E. Mixed red meat dishes cooked with no fat<br>4F. Mixed red meat dishes cooked with olive oil<br>4G. Mixed red meat dishes cooked with butter, margarine, cream<br>4H. Mixed red meat dishes cooked with other fats<br>All red meat (pork, lamb, beef, veal, meat products, including mixed dishes, homemade, retail, ready meals, take-away)	'4D' (g) + '4E' (g) + '4F' (g) + '4G' (g) + '4H' (g)<br>Total red meat (servings) = Total red meat (g)/125<br><br>1 point: Total red meat (servings) <1
6. How many servings of butter, margarine, or cream do you consume per day? *(1 serving = 12g, 1 tablespoon)*	Less than 1	5. Butter, margarine and cream (all types of butter, margarine and fat spreads, and cream, including whipped), and:<br>2C. Salad and raw vegetables with butter, margarine, cream<br>2G. Vegetables cooked with butter, margarine, cream<br>2K. Mixed vegetable dishes cooked with butter, margarine, cream<br>4C. Red meat cooked with butter, margarine, cream<br>4G. Mixed red meat dishes cooked with butter, margarine, cream<br>8C. Pulses cooked with butter, margarine, cream<br>8G. Mixed pulse dishes cooked with butter, margarine, cream<br>9C. Fish cooked with butter, margarine, cream<br>9G. Mixed fish dishes cooked with butter, margarine, cream<br>9K. Shellfish/seafood cooked with butter, margarine, cream	Total butter, margarine, cream (g) = '5' (g) + '2C (g) + '2G' (g) + '2K' (g) + '4C' (g) + '4G' (g) + '8C' (g) + '8G' (g) + '9C' (g) + '9G' (g) + '9K' (g) + '9O' (g) + '12C (g) + '14C' (g)<br>Total butter, margarine, cream (servings) = Total butter, margarine, cream (g)/12<br><br>1 point: Total butter, margarine, cream (servings) <1

		9O. Mixed shellfish/seafood dishes cooked with butter, margarine, cream<br>12C. Poultry and rabbit dishes cooked with butter, margarine, cream<br>14C. Miscellaneous foods/meals cooked with butter, margarine, cream	
7. How many sugar-sweetened beverages do you drink per day? *(1 cup = 100 ml)*	Less than 1 cup	6. All sugary soft drinks (whether carbonated or not, e.g. squash, cordial, soda, energy drinks). Does not include coffee and other warm beverages containing sugar	Total sugar-sweetened beverages (ml) = ‘6’ (ml)<br>Total sugar-sweetened beverages (servings) = Total sugar-sweetened beverages (ml)/100<br><br>1 point: Total sugar-sweetened beverages (servings) <1
8. How much wine do you drink per week? *(1 glass = 125 ml)*	7 or more glasses	7. Wine (red, white, rose, including champagne and prosecco types)	Total wine (ml) = ‘7’ (ml)<br>Total wine (servings) = Total wine (ml)/125<br><br>Total wine (servings/week) = Total wine (servings)/3 *7<br><br>1 point: Total wine (servings/week) ≥7
9. How many servings of pulses do you consume per week? *(1 serving = 150g) (including canned varieties)*	3 or more	8A. Pulses cooked with no fat<br>8B. Pulses cooked with olive oil<br>8C. Pulses cooked with butter, margarine, cream<br>8D. Pulses cooked with other fats<br>8E. Mixed pulse dishes cooked with no fat	Total pulses (g) = ‘8A’ (g) + ‘8B’ (g) + ‘8C’ (g) + ‘8D’ (g) + ‘8E’ (g) + ‘8F’ (g) + ‘8G’ (g) + ‘8H’ (g)<br>Total pulses (servings) = Total pulses (g)/150<br><br>Total pulses (servings/week) = Total pulses

		8F. Mixed pulse dishes cooked with olive oil<br>8G. Mixed pulse dishes cooked with butter, margarine, cream<br>8H. Mixed pulse dishes cooked with other fats<br>All pulses (including mixed dishes, homemade, retail, ready meals, take-away, baked beans)	(servings)/3 *7<br><br>1 point: Total pulses (servings/week) ≥3
10. How many servings of fish or shellfish/seafood do you consume per week? *(1 serving = 100-150 g fish, or 4-5 pieces or 200 g shellfish)*	3 or more	9A. Fish cooked with no fat<br>9B. Fish cooked with olive oil<br>9C. Fish cooked with butter, margarine, cream<br>9D. Fish cooked with other fats<br>9E. Mixed fish dishes cooked with no fat<br>9F. Mixed fish dishes cooked with olive oil<br>9G. Mixed fish dishes cooked with butter, margarine, cream<br>9H. Mixed fish dishes cooked with other fats<br>9I. Shellfish/seafood cooked with no fat<br>9J. Shellfish/seafood cooked with olive oil<br>9K. Shellfish/seafood cooked with butter, margarine, cream<br>9L. Shellfish/seafood cooked with other fats<br>9M. Mixed shellfish/seafood dishes cooked with no fat<br>9N. Mixed shellfish/seafood dishes cooked with olive oil<br>9O. Mixed shellfish/seafood dishes cooked with butter, margarine, cream<br>9P. Mixed shellfish/seafood dishes cooked with other fats<br>All white and oily fish and shellfish/seafood (including mixed dishes, homemade, retail, ready meals, take-away)	Fish:<br>Total fish (g) = '9A' (g) + '9B' (g) + '9C' (g) + '9D' (g) + '9E' (g) + '9F' (g) + '9G' (g) + '9H' (g)<br>Total fish (servings) = Total fish (g)/125<br><br>Shellfish/seafood:<br>Total shellfish/seafood (g) = '9I' (g) + '9J' (g) + '9K' (g) + '9L' (g) + '9M' (g) + '9N' (g) + '9O' (g) + '9P' (g)<br>Total shellfish/seafood (servings) = Total shellfish/seafood (g)/200<br><br>Total fish and shellfish/seafood:<br>Total fish and shellfish/seafood (g) = Total fish (g) + Total shellfish/seafood (g)<br>Total fish and shellfish/seafood (servings) = Total fish (servings) + Total shellfish/seafood (servings)<br><br>Total fish and shellfish/seafood (servings/week) =

			Total fish and shellfish/seafood (servings)/3 *7<br><br>1 point: Total fish and shellfish/seafood (servings/week) ≥3
11. How many times per week do you consume commercial sweets or pastries (not homemade), such as cakes, cookies, biscuits, or custard?	Less than 2	10. Commercial sweets and pastries (including all non-homemade sweets and bakery goods)	Frequency of consumption of commercial sweets and pastries (times/3 days)<br>Frequency of consumption of commercial sweets and pastries (times/week) = Frequency of consumption of commercial sweets and pastries (times/3 days)/3 *7<br><br>1 point: Frequency of consumption of commercial sweets and pastries (times/week) <2
12. How many servings of nuts (including peanuts) do you consume per week? *(1 serving = 30g)*	3 or more	11. Nuts (all types of salted and unsalted nuts and nut butters, eaten alone or in mixed dishes)	Total nuts (g) = '11' (g)<br>Total nuts (servings) = Total nuts (g)/30<br>Total nuts (servings/week) = Total nuts (servings)/3 *7<br><br>1 point: Total nut (servings/week) ≥3
13. Do you prefer to eat chicken, turkey, or rabbit meat instead of beef, pork, hamburgers, or sausages?	Yes = 1 point	12A. Poultry and rabbit dishes cooked with no fat<br>12B. Poultry and rabbit dishes cooked with olive oil<br>12C. Poultry and rabbit dishes cooked with butter, margarine, cream<br>12D. Poultry and rabbit dishes cooked with other fats<br>All poultry (chicken, turkey, duck) and rabbit, including mixed	Total poultry and rabbit (g) = '12A' (g) + '12B' (g) + '12C' (g) + '12D' (g)<br>Total red meat (g) ('4A' (g) + '4B' (g) + '4C' (g) + '4D' (g) + '4E' (g) + '4F' (g) + '4G' (g) + '4H' (g)<br><br>1 point: Total poultry and rabbit (g) > Total red meat

		dishes, homemade, retail, ready meals, take-away)	(g)
14. How many times per week do you consume cooked vegetables, pasta, rice, or other dishes prepared with a sauce of tomato, garlic, onions or leeks sautéed in olive oil (sofrito)?	2 or more = 1 point	13. Tomato sauce with garlic, onions or leeks and other condiments sautéed in olive oil	Frequency of consumption of sofrito (times/3 days)<br>Frequency of consumption of sofrito (times/week) = Frequency of consumption of sofrito (times/3 days)/3 *7<br><br>1 point: Frequency of consumption of sofrito (times/week) ≥2
